# Physical activity patterns, adherence to using a wearable activity tracker during a 12-week period and correlation between self-reported function and physical activity in working age individuals with hip and/or knee osteoarthritis

**DOI:** 10.1186/s12891-021-04338-x

**Published:** 2021-05-15

**Authors:** Elin Östlind, Anita Sant’Anna, Frida Eek, Kjerstin Stigmar, Eva Ekvall Hansson

**Affiliations:** 1 Department of Health Sciences, Division of Physiotherapy, Lund University, Lund, Sweden; 2 Dalby healthcare center, Region Skåne Lund, Sweden; 3 Viniam Consulting AB, Halmstad, Sweden; 4 Skåne University Hospital, Lund, Sweden

**Keywords:** Hip osteoarthritis, Knee osteoarthritis, Physical activity, Wearable activity tracker, Mhealth, Fitbit

## Abstract

**Background:**

A majority of individuals with osteoarthritis (OA) are insufficiently physically active. Self-monitoring with wearable activity trackers (WAT) could promote physical activity (PA), and increased knowledge of PA patterns and adherence to using a WAT is needed. The aim of this study was to describe PA patterns and adherence to WAT-use during an intervention among participants of working age with hip and/or knee OA. The study further explores the correlation between self-reported joint function and PA.

**Methods:**

Individuals of working age with hip and/or knee OA who used a WAT, Fitbit Flex 2, for 12 weeks were included. Participants monitored their PA in the Fitbit-app. An activity goal of 7,000 steps/day was set. Steps and minutes in light (L), moderate and vigorous (MV) PA were collected from the Fitbit. Self-reported joint function (HOOS/KOOS) was completed. Data was analyzed with linear mixed models and Spearman’s rank correlation.

**Results:**

Seventy-five participants (45–66 years) walked on average 10 593 (SD 3431) steps/day, spent 248.5 (SD 42.2) minutes in LPA/day, 48.1 (SD 35.5) minutes in MVPA/day, 336.0 (SD 249.9) minutes in MVPA/week and used the Fitbit for an average of 88.4 % (SD 11.6) of the 12-week period. 86.7 % took > 7,000 steps/day and 77.3 % spent > 150 min in MVPA/week. Mean daily steps/week decreased significantly over the 12 weeks (β-coefficient − 117, 95 % CI -166 to -68, *p* = < 0.001) as well as mean daily minutes in LPA/week (β-coefficient − 2.3, 95 % CI -3.3 to -1.4, *p* = < 0.001), mean daily minutes in MVPA/week (β-coefficient − 0.58, 95 % CI -1.01 to -0.16, *p* = 0.008) and mean adherence to Fitbit-use per week (β-coefficient − 1.3, 95 % CI -1.8 to -0.8, *p* = < 0.001). There were no significant correlations between function (HOOS/KOOS) and PA.

**Conclusions:**

The majority of participants reached 7,000 steps/day and the recommended 150 min in MVPA per week. However, PA decreased slightly but gradually over time. Adherence to using the Fitbit was high but also decreased during the intervention. Understanding PA patterns and the use of a Fitbit to promote PA could be beneficial in tailoring interventions for individuals with hip and/or knee OA.

## Background

Osteoarthritis (OA) is one of the most common musculoskeletal disorders in the world [1], causing pain [2], disability and reduced quality of life [3]. OA can affect any synovial joint, but the knee, hip and hand are the most common sites [4]. Knee OA is more common in women and the prevalence levels out at 50 years of age while hip OA continues to increase with age [5]. The prevalence of physician-diagnosed hip and knee OA has been estimated as 5.8 and 13.8 % respectively in a large population ≥ 45 years in Sweden [4]. OA represents a substantial burden for the individual as well as society due to increased work absenteeism and healthcare costs [3]. Many individuals with OA have one or several comorbidities such as hypertension, dyslipidemia and diabetes [6]. Studies have also shown an increased risk of cardiovascular disease [7], and death due to cardiovascular disease in individuals with OA compared to individuals without OA [8].

The recommended first-line treatment for OA is physical activity (PA); it reduces pain, improves physical function and health-related quality of life for individuals with lower limb OA [9]. The World Health Organization (WHO) defines PA as “any bodily movement that requires energy expenditure” whereas exercise is defined as “a subcategory of PA that is planned, structured, repetitive and aims to improve or maintain one or more components of physical fitness.” [10]. Previous research has shown that aquatic or land-based exercise as well as strength training, aerobic exercise or tai chi were associated with reduction in pain and improvement in physical function and health-related quality of life [9]. Moderate intensity PA for at least 150 min per week or 75 min of vigorous intensity PA per week or a combination of both moderate and vigorous PA (MVPA) is recommended to all adults by the WHO to reduce the risk of all-cause mortality, coronary heart disease, type 2 diabetes, depression and several other diseases [11]. These recommendations have also been converted to steps per day and correspond to approximately 7,100 − 11,000 steps per day [12]. However, a majority of individuals with hip and/or knee OA do not meet these PA recommendations [13, 14].

One reason for persons not meeting PA recommendations could be that individuals with OA often experience pain and reduced function in affected joints [15]. This has been associated with lower level of PA [16–18] whereas higher levels of PA have been associated with higher self-reported and objective functional measures in older adults with or at risk for OA [19]. Veenhof et al. [18] concluded that individuals with low physical function need additional stimulation from clinicians to be more physically active. Low physical function may predict level of PA and it is therefore important to explore the correlation between self-reported function and PA in an OA-population but studies on individuals of working age (18–67 years) are lacking.

Treatment strategies that include self-management skills to support the maintenance of PA, rather than supervised exercise, may be cost-effective and important in the treatment of OA [20, 21]. Behavioral change techniques (BCTs) can improve self-management skills and are used in interventions to increase adherence to exercise. Patient led goal-setting, self-monitoring of behavior and non-specific rewards are examples of BCTs that are effective in improving adherence to PA in both short and long-term [22]. A relatively novel and popular method that applies BCTs and has shown to increase PA in healthy populations as well as in populations with OA and other chronic conditions is the utilization of wearable activity trackers (WATs) [23–25]. WATs are sensors that track movement and can be paired with a smartphone, tablet or computer application (App). A WAT can be worn on different locations, most commonly on the wrist, and are manufactured by large companies such as Fitbit, Jawbone, Apple, Polar and Nike. WATs have gained increased attention from the general population as well as researchers and clinicians during the last decade with a twenty-fold increase in number of WAT-studies, from 8 to 2013 to 199 in 2017 [26].

WATs have been suggested as a complement to traditional short-term exercise interventions to increase the long-term adherence to PA-participation [23]. The concept adherence has been defined by the WHO as “the extent to which a person’s behavior – taking medication, following a diet, and/or executing lifestyle changes, corresponds with agreed recommendations from a healthcare provider” [27]. For home exercises, a high adherence is associated with better outcomes on pain, physical function and self-perceived effect in patients with hip or knee OA but the adherence to exercise decreases over time [28]. Adherence to using a WAT in a PA-intervention has been analyzed by a few studies in patients with rheumatic and musculoskeletal diseases [25, 29, 30], including knee osteoarthritis [31] and in breast cancer [32, 33]. Different definitions of adherence were used in these studies but overall, they showed a high short-term adherence to using the WAT. To our knowledge, only one study so far has explored adherence to WAT use in individuals with OA, but that was in combination with a novel app (OA GO) [31]. Studies describing PA patterns during a PA-intervention and adherence to using a WAT in individuals of working age (18–67 years) with OA are lacking.

Hip and knee OA is common also in the working population, affecting physical function, work ability, and increasing the risk of comorbidities. Consequently, it is important to conduct studies on individuals of working age to describe and explore interventions that can improve function, work ability and prevent comorbidities. The aim of this study was to describe PA patterns and adherence to using a WAT, Fitbit Flex 2, among individuals of working age with hip and/or knee OA, during a 12-week period. A secondary aim was to explore the correlation between baseline self-reported function and subsequent PA.

## Methods

### Design

In this exploratory study, we analysed WAT-data from Fitbit Flex 2 (Fitbit Inc., San Francisco, CA, USA) during 12 weeks. The data was collected during a cluster-randomized trial (C-RCT) (Clinical trials No: NCT03354091) [34], in which treatment as usual (participating in a Supported Osteoarthritis Self-management Program, SOASP) [35, 36] was compared with treatment as usual together with self-monitoring PA using a commercial WAT, Fitbit Flex 2, for 12 consecutive weeks as an add-on. This study reports PA data on the intervention group (*n* = 80) only.

### Setting

The setting for this study is the Supported Osteoarthritis Self-management Program (SOASP) [35, 36] in primary care. The SOASP offers first line treatment for patients with hip, knee and/or hand OA and is offered on a regular basis in primary health care. The minimal intervention in the SOASP is two theoretical group sessions including information about OA, exercise and self-management held by a physiotherapist (PT). After the theoretical sessions, patients are offered an individual appointment with a PT and are introduced to specific exercises based on the patient’s needs and goals. Some healthcare centers or physiotherapy clinics offer supervised group training, often for a limited period, e.g. two times a week for six weeks.

### Participants and recruitment

This current study included eighty participants from a C-RCT that had used a Fitbit and Fitbit-app to monitor PA for 12 weeks. Three participants had technical issues and dropped out during the first weeks of the intervention. Two of the remaining participants completed the intervention but their data was lost due to technical issues when transferring data to the project’s server. Consequently, we finally included participants (*n* = 75) in the intervention group of the C-RCT that had > 50 % data from the Fitbit. Eligible for recruitment in the C-RCT were individuals of working age in southern Sweden with hip and/or knee OA. The inclusion criteria for the C-RCT were: working ≥ 50 % (20 h. /week), aged between 18 and 67 years, being able to understand Swedish in speech and writing and able to walk and participate in some form of exercise. They also had to have access to a smartphone, tablet or computer to use the Fitbit-app and be able to wear a WAT for 12 weeks.

 Potential participants for the C-RCT were approached in two different ways; from SOAPS offered on a regular basis at different health care centers and from advertisement. PTs at healthcare centers and physiotherapy clinics in southern Sweden were contacted in 2017–2018 and asked to inform individuals participating in the SOASP about the research project. Participants in the SOASPs were given oral and written information about the research project, inclusion criteria and how to register. They received this information from EÖ or the PT that held the theoretical sessions. The individuals self-registered on the project’s website using an electronic identification (ID) service [37] and thereby gave their informed consent. Before being able to register, they were asked if they met the inclusion criteria in the study. Participants from each SOASP were cluster-randomized using sealed envelopes to control or intervention. After a year of recruiting individuals, a Facebook advertisement was added to recruit additional participants in 2018–2019. The advertisement targeted individuals living within a defined geographical area in southern Sweden aged between 40 and 67 years. The age range was pragmatically chosen to limit the number of people unnecessarily exposed to the advertisement, since few individuals < 40 years have OA. Individuals that were interested e-mailed EÖ and then received more information about the research project, inclusion criteria and how to register. Individuals that registered on the project’s website took part of a SOASP offered within the research project. EÖ was responsible for the SOASPs held within this project that consisted of three theoretical sessions. An individual visit with a PT (EÖ) was also offered. Each SOASP was cluster-randomized exactly as the other SOASPs. Twenty-two individuals were recruited from SOASPs at health care centers and 53 were recruited with Facebook advertisement.

### Self-monitoring PA with Fitbit

The participants self-monitored their PA with a wrist-worn WAT, the Fitbit Flex 2. In connection to participating in the SOASP, each participant met with EÖ and received the Fitbit. They were aided in installing the Fitbit application (app), synchronizing the device to their app as well as connecting their FitBit account to the study via the project’s website. The participants were asked to wear the Fitbit for 12 weeks, from morning until bedtime. The intervention length of 12 weeks was chosen to allow for a true change of PA-behavior and potential health benefits to accrue. In a previous study, Lally et al. [38] reported a median of 66 days to automatize a new behavior. In this study, the participants were also asked to monitor their activity by using the app once a day. Using the app once per day allowed for synchronization of the data from the device to the app. During the 12-week period, there were no planned reminders but if participants had several [4, 5] days without registered activity, EÖ contacted them by e-mail to ensure that there were no technical issues. The default activity goal for the Fitbit of 10,000 steps per day was changed to 7,000 in order to make it more achievable for this population with hip and/or knee OA. Previous research has also suggested that 7,000 steps per day might be an accurate estimate for meeting the recommended 150 min per week of MVPA [12, 39]. There were also other default activity goals in the app; *distance* (8.05 km), *calories burned* (based on gender and weight) and bouted *active minutes* (30 min). Participants were asked not to change them.

### Outcomes and measurements

In this current study, we defined *PA patterns* as daily steps and minutes in LPA and MVPA per day distributed during the Fitbit-use period of 12 weeks. We defined *adherence* as the total percentage of Fitbit-use during the 12 weeks. The outcomes in this study were PA patterns during 12-week Fitbit-use presented as average daily number of steps per week and average daily minutes in LPA and MVPA per week. Additional outcomes were adherence to using the Fitbit, average total weekly MVPA and self-reported function. PA was monitored by Fitbit Flex 2, a commercial accelerometer-based WAT that continually estimates steps taken, distance traveled, and time in different activity levels. The Fitbit is waterproof and can be worn during swimming and showering. The device is worn inside a rubber wristband and has five small LED-lights but no display. The measurements are transmitted via Bluetooth from the device to a smartphone, tablet or computer app, which in turn transfers the data to the Fitbit servers. All registered activity data can be viewed anytime on the app or on the Fitbit user portal. Two systematic reviews on validity and reliability of commercial WATs has shown an overall high validity for steps and, to a lesser extent, duration of PA [40, 41]. The reliability and validity of the Fitbit Flex and Fitbit Flex 2 have been evaluated by a few studies but the results are inconsistent. A majority of the studies reported that Fitbit Flex overestimated number of steps and time in MVPA compared to actigraph, which is commonly used as a ‘gold standard’ for measuring PA in free-living setting [42–44].

In this study, we considered only the number of steps and the time spent in LPA and MVPA per day as estimated by the Fitbit. Fitbit uses a proprietary algorithm to estimate metabolic equivalents (METs). One MET is described as the energy expended during rest or sitting quietly. An activity is considered moderate or vigorous if it continues for > 10 min and exceeds 3 METs and 6 METs respectively [45, 46]. Activities above rest and below moderate PA are considered light (L) PA. The Fitbit-measured activity data is made available to third parties via Fitbits Web APIs [47]. We developed our own data server, which would query the Fitbit’s Web API every evening for activity from our study participants during the previous day. In particular, we requested number of steps and number of minutes in light, moderate and vigorous activity for every minute of the previous day. Distance and sedentary time were also collected but not analyzed in this study. In order to be able to query the Fitbit Web APIs we submitted a request to Fitbit and obtained approval for our research study. We requested that participants grant us access to their Fitbit accounts via our project website when they received the Fitbit from the researcher who monitored the study (EÖ).

Subjective function was measured by the Hip injury and Osteoarthritis Outcome Score (HOOS) and Knee injury and Osteoarthritis Outcome Score (KOOS) [48, 49]. HOOS/KOOS has five subscales, each containing 2–17 items: Pain, Symptoms, Activities of Daily Living (ADL), Sport and Recreation function (Sport/Rec) and hip/knee-related Quality of Life (QoL). Each subscale is calculated independently as the mean score of the individual items in the subscale. The score goes from 0 to 100 in which zero indicates extreme hip/knee problems and 100 indicates no hip/knee problems. HOOS/KOOS have been shown to have adequate psychometric properties for individuals with OA [48–51]. In this study, participants filled out either HOOS or KOOS at baseline depending on their most affected joint.

### Analysis of PA

The raw activity data from the Fitbit was pre-processed using Rstudio [52] and Microsoft Corporation, Microsoft Excel. 2018. The subsequent statistical analysis was undertaken in IBM SPSS Statistics for Windows, version 25 (IBM Corp., Armonk, N.Y., USA). The data from each participant was identified with an anonymous random string. We included data from days 2–85 in the analysis. Day 1 was excluded because, in most cases, participants only started using the Fitbit in the afternoon. All days after day 85 were excluded because we wanted to analyze twelve full weeks (84 days). *Valid days* to be included in analyses of PA pattern were defined as days with > 1,500 steps [53, 54]. Days with < 1500 steps were defined as “missing”. If there were ≥ four *missing days* in one week, the whole week was excluded from the analysis. For each participant, each calendar day was characterized by the total number of steps, total number of minutes in LPA and total number of minutes in MVPA. The mean number of steps, minutes in LPA and minutes in MVPA were calculated for each of the twelve weeks for each participant. The total number of minutes in MVPA for each week were also calculated for each participant. If one to three days in a week were missing, the number of minutes in MVPA for the missing days was imputed as the mean value of the remaining days that week. This was done so that the total number of minutes in MVPA could be estimated for that week.

Mean (SD) number of steps, minutes in LPA and MVPA per day, as well as per week and total minutes in MVPA per week for the entire period are presented.

### Analysis of adherence

For each participant, adherence to Fitbit-use during the entire period was calculated and presented as the percentage of *valid* days (> 1,500 steps) during the study period. The adherence to Fitbit-use per week was also calculated by dividing the number of *valid days* per week by seven (days in a week). Eight participants did not have twelve full weeks and consequently, their last week of intervention had zero to six days. Weeks with ≤ four days were excluded and in weeks with ≥ four days, the adherence to Fitbit-use was calculated by dividing the number of *valid days* with the number of days in that week (four to six). For the whole group, adherence to Fitbit-use was calculated as mean of the percentage of adherence for the entire period and for each week.

### Statistical analysis

To explore PA patterns and adherence to using the Fitbit during the 12-week intervention, we carried out a linear mixed model with a random intercept to account for the weeks clustered within each participant. The dependence between repeated measurements was modelled using an autoregressive covariance structure (AR[1]). The linear trend (β-coefficient) represents the change in each outcome variable per week during the total 12-week period. Correlations between baseline function (each HOOS/KOOS subscale) and mean number of steps, minutes in LPA and MVPA/day during the 12 weeks were assessed by Spearman’s rank correlation. Significance level was set to 0.05.

## Results

### Participants

Seventy-five individuals (10 men, 65 women) aged 45 to 66 years with hip and/or knee OA were included in this study. Seventeen individuals rated the hip as the most affected joint and filled out HOOS whereas 58 individuals rated the knee as the most affected joint and filled out KOOS. One individual did not fill out the questionnaire at baseline, characteristics are therefore presented for 74 individuals (Table 1).

**Table 1:** Participant characteristics and baseline data (HOOS/KOOS) (*n* = 74)

**Age (years), mean (SD)**	56.9 (5.2)
**Gender (female), n (%)**	64 (86.5)
**Married or living with partner, n (%)**	56 (75.7)
**Most affected joint, n (%)**	
Hip	17 (22.7)
Knee	58 (77.3)
**Education (postsecondary), n (%)**	49 (66.2)
**Employment (percentage of full time – 40 h.) n (%)**	
0–25 %	2 (2.7)
26–50 %	5 (6.8)
51–75 %	8 (10.8)
76–100 %	58 (78.4)
Unemployed	1 (1.4)
**Physically demanding work, n (%)**	
No	52 (70.3)
Yes, several times a week	7 (9.5)
Yes, daily	14 (18.9)
Missing	1 (1.4)
** Sedentary work, sitting > 50 %, n (%)**	39 (52.7)
Missing	2 (2.7)
**Current self-assessed PA-level compared to PA-level before OA, n (%)**	
More physically active	8 (10.8)
Equally physically active	41 (55.4)
Less physically active	24 (32.4)
Missing	1 (1.4)
**Regular usage of a WAT during the last three months before the intervention, n (%)**	
Yes	29 (39.2)
No	41 (55.4)
Missing	4 (5.4)
**HOOS (*n* = 17), mean (SD)**	
Pain	46.8 (18.2)
Symptoms	58,3 (22)
ADL	63.1 (22.7)
Sport/Rec	39.2 (22.8)
QoL	40.6 (18.4)
**KOOS (*n* = 58), mean (SD)**	
Pain	60.6 (19.7)
Symptoms	52.2 (20.4)
ADL	72 (19.5)
Sport/Rec	27.5 (25.5)
QoL	43.8 (19.1)

*HOOS* Hip injury and Osteoarthritis Outcome Score; *KOOS* Knee injury and Osteoarthritis Outcome Score; *SD * Standard deviation; *PA* Physical activity; *OA* Osteoarthritis; *WAT* Wearable activity tracker; *ADL* Activities of daily living; *QoL* Quality of life

### PA patterns during 12-weeks Fitbit-use

**Fig. 1:**
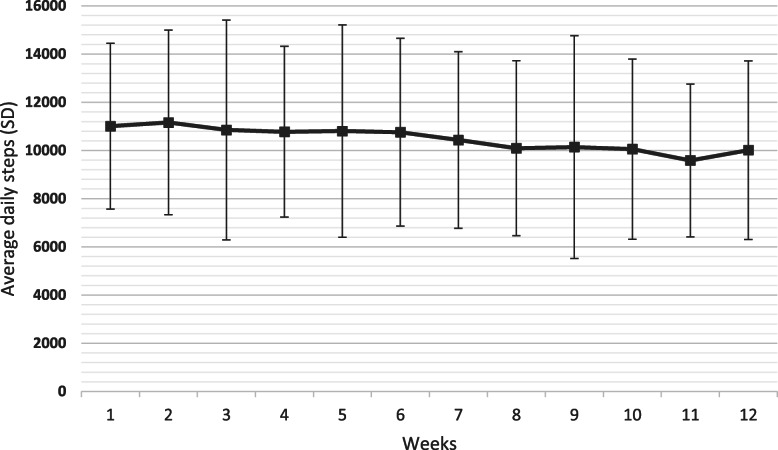
Mean (SD) daily step counts across all participants for each week during the 12 week intervention (*n* = 75).

**Fig. 2:**
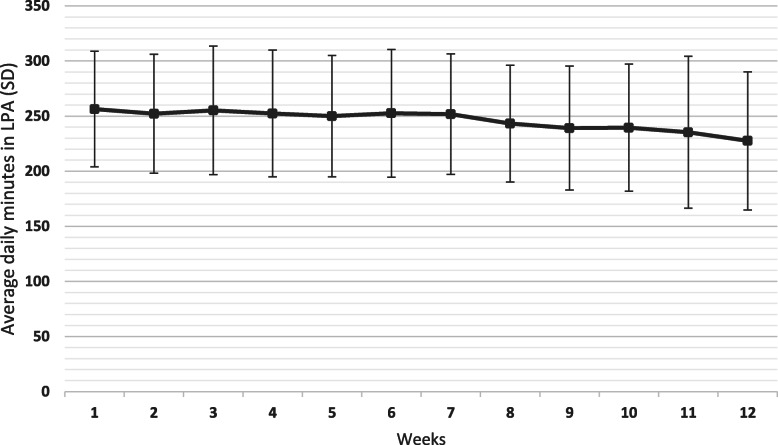
Mean (SD) daily minutes in light physical activity (LPA) across all participants for each week during the 12 week intervention (*n* = 75).

**Fig. 3:**
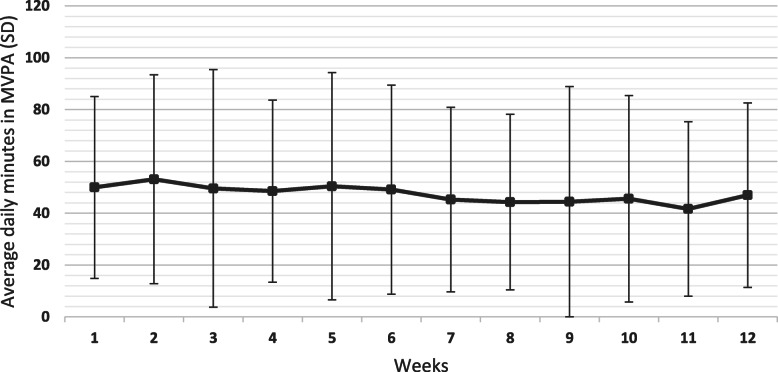
Mean (SD) daily minutes in moderate-to-vigorous physical activity (MVPA) across all participants for each week during the 12 week intervention (*n* = 75).

The participants took 10 593 (SD 3431) number of steps per day during the 12-week period, spent 248.5 (SD 42.2) minutes in LPA and 48.1 (SD 35.5) minutes in MVPA per day and 336.0 (SD 249.9) minutes in MVPA per week. There was a slight gradual statistically significant decrease in mean number of daily steps per week over the 12 weeks (β-coefficient − 117, 95 % CI -166 to -68, *p* = < 0.001) (Fig. 1). Week 2 had the highest average number of steps per day with 11 162 (SD 3830) steps, and week 11 the lowest number of steps with 9589 (SD 3169) steps per day. Daily minutes in LPA per week also decreased slightly but statistically significant over the 12 weeks (β-coefficient − 2.3, 95 % CI -3.3 to -1.4, *p* = < 0.001) (Fig. 2) as well as daily minutes in MVPA per week (β-coefficient − 0.6, 95 % CI -1.01 to -0.16, *p* = 0.008) (Fig. 3). The highest number of daily minutes in LPA per week was 256.4 min in week 1 and the lowest was in week 12 with 227.6 daily minutes per week. For MVPA, the highest daily minutes per week was in week 2 with 53.1 min, and the lowest in week 11, 41.7 min. Sixty-five (86.7 %) participants had on average > 7000 steps per day, 35 (46.7 %) participants had on average > 10 000 steps per day and 58 (77.3 %) had on average > 150 min in MVPA per week.

### Patterns of adherence to using the Fitbit during 12 weeks

**Fig. 4:**
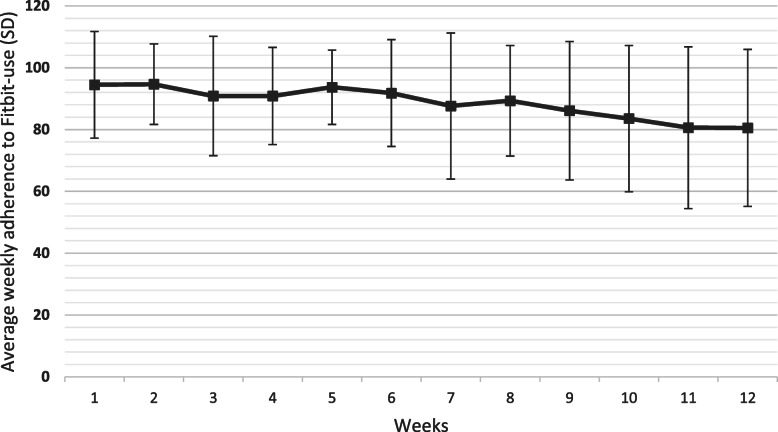
Mean weekly adherence to Fitbit-use across all participants for each week during the 12 week intervention (*n* = 75)

Sixty-seven participants completed the maximum number of days (84) whereas eight participants finished the intervention prematurely due to practical and technical reasons. They participated during a total number of days of 63–83 days with a mean value of 73 days. The Fitbit was used on average 88.4 % (SD 11.6) of the days during the 12-week intervention with highest adherence in week 2 (94.7 %) and lowest in week 12 (80.5 %). The adherence to Fitbit-use decreased gradually over the 12 weeks (β-coefficient − 1.3, 95 % CI -1.8 to -0.8, *p* = < 0.001) (Fig. 4).

### Correlation between baseline function and subsequent PA

No correlations were found between HOOS/KOOS subscales at baseline and subsequent PA (average number of steps/day and minutes in LPA and MVPA/day during the 12 weeks) (Table 2).

**Table 2:** Correlation (Spearman’s Rho) between HOOS/KOOS subscales and average number of steps, minutes in LPA and MVPA per day (*n* = 74)

	**Steps/day**	**p-value**	**LPA/day**	**p-value**	**MVPA/day**	***p*-value**
**Pain**	0.061	0.605	0.020	0.867	0.029	0.807
**Symptoms**	0.027	0.818	-0.083	0.484	-0.001	0.992
**ADL**	0.108	0.360	0.014	0.907	0.054	0.649
**Sport/Rec**	0.195	0.103	0.153	0.203	0.094	0.434
**QoL**	0.123	0.301	0.188	0.112	0.064	0.592

## Discussion

This study provides insights into Fitbit-measured PA patterns and adherence to using a Fitbit in individuals of working age with hip and/or knee OA during a 12-week intervention. We included only participants with > 50 % Fitbit data. The vast majority of the participants were highly active and reached on average the recommended minimum of > 150 min in MVPA per week and the activity goal in the Fitbit-app > 7000 steps per day. The adherence to using the Fitbit was also high during the 12 weeks. A significant but small decrease was seen in both PA and adherence to using the Fitbit during the period. There were no significant correlations between baseline function as measured with HOOS/KOOS and subsequent PA.

The participants were highly active throughout the intervention, even in the least active week, week 11, they walked on average almost 10,000 steps (9,589) per day and had 305 total minutes in bouted MVPA, twice the amount of the recommended minimum of 150 min per week [11]. This study reports higher levels of PA compared to previous studies with hip or knee OA participants [14, 39, 55–57]. A systematic review showed that participants with hip and knee OA averaged 8,174 respectively 7,753 steps per day and 160 respectively 50 min of MVPA (in bouts of ≥ 10 min) per week [39]. A more recent study of a US population with or at risk of knee OA reported similar results with 17.2 % men and 9.2 % women meeting the recommended level of ≥ 150 min a week of bouted MVPA [14] which is in contrast to this study with 77.3 % of participants reaching ≥ 150 min per week of bouted MVPA. However, these results can be difficult to interpret due to differences in measurement. The most common accelerometer used in these studies was the non-commercial accelerometer Actigraph that is usually worn on the hip [14, 39]. A few studies have used a Fitbit to measure PA in OA-populations as an outcome measure and they also reported markedly lower number of steps per day compared to our study [55–57]. A possible explanation for this could be differences in participant characteristics. In our study, the participants were younger and had better joint function (as measured with HOOS/KOOS) than the participants in the previous studies using Fitbit. Furthermore, the high levels of PA in this study could also be explained by the fact that participants in this study had taken part of the SOASP and received information about the importance of exercise. The activity goal and continuous feedback from the Fitbit could also have affected the participants PA-levels.

The analyses of the activity data in this study showed a trend of a significant but small decrease of 117 number of daily steps per week and 0.6 daily minutes in MVPA per week. This small decrease in PA may not be clinically important and in week 12, participants were still highly active. However, the decrease could indicate that it is difficult to change and maintain behavior and that PA-interventions often prove to be most effective in the short-term [58]. Hartman et al. [33] also analyzed the weekly PA-pattern in an intervention using a Fitbit. In their study, MVPA per week also differed significantly but there was a slight increase in MVPA during the 12-week intervention, which is in contrast to the results in our study. One explanation for this could be the differences in follow-ups and reminders. Participants in that study received several planned phone calls and e-mails during the intervention [32].

Adherence to using the Fitbit was on average high throughout the 12 weeks but decreased slightly and gradually. To charge and wear the device is a prerequisite for the utilization of WATs to be an effective method to increase and optimize PA [59]. The average adherence of 88 % in this study is comparable with the adherence presented in a recent systematic review, on participants with rheumatic and musculoskeletal diseases, in which three studies using wrist-worn WATs for 12 weeks reported an average adherence of 93 % [25, 29–31]. In two of the three studies, participants had several follow-ups, which might have affected the adherence to using the WATs [30, 31]. In studies on other populations with longer use of the Fitbit (9 and 12 months) and no follow-ups, the adherence was lower and had a gradual decrease. In the 9-month study, the overall adherence was only 44.5 % and the authors concluded that participants who stopped wearing the device did not return to compliance on their own. They also reported that participants with positive attitudes toward technology had higher adherence to using the Fitbit [32]. In the 12-month study, 50 % used their Fitbit after six months and after 300 days, only 12 % still used it. The most common reasons for participants to stop using the Fitbit were technological failures and empty batteries [60]. In our study, we did not have any scheduled reminders to use the Fitbit but the contact that was made with some participants lacking data for several days could also be a reminder to use the Fitbit. If no contact had been taken with the participants, the adherence in this study might have been lower.

This study also explored the correlation between baseline function measured with HOOS/KOOS and the subsequent PA measured with the Fitbit. Rosemann et al. [16] found that two of the main factors associated with lower levels of self-reported PA in participants with hip or knee OA were dysfunction in the lower limb and pain. If a lower score is associated with lower subsequent PA, this could help clinicians to identify individuals with OA that are in need of extra support to maintain/increase PA-levels. Heiberg & Figved [61] reported that individuals who preoperative scored lower on HOOS subscales Pain and Sport/Rec were at risk of being less physically active in the long-term after a total hip arthroplasty. However, the results in this study showed weak and non-significant correlations between the subscales on HOOS/KOOS and level of PA [62]. A reason for this might be that the participants in this study were more active and had overall better function and less pain as measured with HOOS/KOOS compared to other OA-populations [13, 14, 51, 61, 63]. The activity data was also collected during a PA-intervention that might have increased their PA compared to their ordinary levels of PA.

This study has some limitations. The first and most important limitation in this study is the recruitment of eligible participants through Facebook, which we suspect has led to a selection bias. Compared with a large cohort including individuals with OA in Sweden that had participated in the SOASP, there were some major differences in patient characteristics in our study [64]. The participants in our study had a lower mean age (57 compared to 67 years) but this could also be because we only recruited individuals of working age. These younger individuals might be more physically active than the general OA-population often represented in other studies. In our study, there was also a higher percentage of female participants (87 % in this study compared to 69 % in the large cohort) but the gender distribution shows a majority of women also in the large cohort. Lastly, there were also a much higher proportion of participants with post-secondary education in our study (66 % compared to 29 %) [64]. Also, almost 40 % of the participants in this study already regularly used a WAT prior to registering to this study which could indicate that they already were highly physically active compared to the general OA-population. The prevalence of WAT-use in the general populations has been reported to be 12.5 respectively 13.86 % in previous studies in the US and the UK [65, 66].

Our findings correspond well to the findings of a previous study by Macridis et al. [67] which reported that using a WAT was associated with being female, below 60 years of age, having a post-secondary education and meeting physical activity guidelines. Using Facebook to recruit participants is a cost-effective and time saving method but leads to an over representation of young, white women [68]. Hence, our results may not be directly generalizable to the general OA-population.

The second limitation is that seven individuals reported that they worked 0–50 % of full time despite that the inclusion criteria was working ≥ 50 %. However, five of these seven participants reported that they worked 25–50 %, which could indicate that they actually worked 50 % and fulfilled the inclusion criteria. The alternatives should have been 25–49 % to accurately identify those working ≥ 50 %. The third limitation is the measurement properties of the Fitbit Flex 2 used in this study. The choice of WAT was determined in consideration of several aspects such as popularity, cost and feasibility to extract data from the manufacturers’ server. Generally, wrist-worn WATs are less accurate than hip-worn WATs in measuring PA but are more user-friendly and therefore lead to higher adherence to usage [25, 69]. In studies examining the measurement properties of the Fitbit Flex 2 and its predecessor, Fitbit Flex, the results are inconsistent but previous studies report that the Fitbit Flex overestimates steps and MVPA in free-living conditions [41–43]. Since Fitbit is a commercial WAT, it uses a proprietary algorithm that only allows researchers access to already processed data and not the actual raw accelerometer data. This limits the interpretations of the data since the thresholds of different activities are unknown. The findings in this study should be seen in light of these limitations, which affect the interpretation and the generalization of the results. With that being said, we do think that this study provides important information about PA patterns during an intervention with the length of the data collection as a major strength.

## Conclusions

In this study, we found that the included individuals of working age with hip and/or knee OA were highly active and had a high adherence to Fitbit-use during a 12-week period. There was however a slight and gradual decrease in both PA and adherence over time. There were no correlations between baseline self- reported function as measured with HOOS/KOOS and subsequent PA. We believe that the findings in this study may provide useful information about PA patterns and adherence to using a Fitbit during a 12-week period of WAT-use in the OA population. The results indicate that it is important to find methods that promote a sustainable level of PA. Understanding PA patterns and the use of a WAT to promote PA could be beneficial in tailoring interventions for individuals with hip and/or knee OA.

Future research should include a more heterogeneous population of individuals with OA, especially individuals that are physically inactive and do not use a WAT regularly.

## Data Availability

The datasets used and analyzed during the current study are available from the corresponding author on reasonable request.

## Abbreviations

OA: Osteoarthritis
PA: Physical activity
WHO: World Health Organization
MVPA: Moderate-to-vigorous physical activity
BCT: Behavior change techniques
WAT: Wearable activity tracker
C-RCT: Cluster-randomized controlled trial
SOASP: Supported osteoarthritis self-management program
PT: Physiotherapist
EÖ: Elin Östlind
MET: Metabolic equivalent
HOOS: Hip injury and Osteoarthritis Outcome Score
KOOS: Knee injury and Osteoarthritis Outcome Score
SD: Standard deviation
